# Geomorphology variables predict fish assemblages for forested and endorheic rivers of two continents

**DOI:** 10.1002/ece3.8300

**Published:** 2021-11-26

**Authors:** Robert Shields, Mark Pyron, Emily R. Arsenault, James H. Thorp, Mario Minder, Caleb Artz, John Costello, Amarbat Otgonganbat, Bud Mendsaikhan, Solongo Altangerel, Alain Maasri

**Affiliations:** ^1^ Aquatic Biology and Fisheries Ball State University Muncie Indiana USA; ^2^ Kansas Biological Survey and the Department of Ecology and Evolutionary Biology University of Kansas Lawrence Kansas USA; ^3^ Department of Civil and Environmental Engineering South Dakota School of Mines & Technology Rapid City South Dakota USA; ^4^ Department of Biology National University of Mongolia Ulaanbaatar Mongolia; ^5^ Institute of Geography and Geoecology Mongolian Academy of Sciences Ulaanbaatar Mongolia; ^6^ Department of Ecosystem Research Leibniz Institute of Freshwater Ecology and Inland Fisheries (IGB) Berlin Germany

**Keywords:** fish assemblages, functional process zones, habitat, hydrogeomorphology, riparian, valley width

## Abstract

Stream fishes are restricted to specific environments with appropriate habitats for feeding and reproduction. Interactions between streams and surrounding landscapes influence the availability and type of fish habitat, nutrient concentrations, suspended solids, and substrate composition. Valley width and gradient are geomorphological variables that influence the frequency and intensity that a stream interacts with the surrounding landscape. For example, in constrained valleys, canyon walls are steeply sloped and valleys are narrow, limiting the movement of water into riparian zones. Wide valleys have long, flat floodplains that are inundated with high discharge. We tested for differences in fish assemblages with geomorphology variation among stream sites. We selected rivers in similar forested and endorheic ecoregion types of the United States and Mongolia. Sites where we collected were defined as geomorphologically unique river segments (i.e., functional process zones; FPZs) using an automated ArcGIS‐based tool. This tool extracts geomorphic variables at the valley and catchment scales and uses them to cluster stream segments based on their similarity. We collected a representative fish sample from replicates of FPZs. Then, we used constrained ordinations to determine whether river geomorphology could predict fish assemblage variation. Our constrained ordination approach using geomorphology to predict fish assemblages resulted in significance using fish taxonomy and traits in several watersheds. The watersheds where constrained ordinations were not successful were next analyzed with unconstrained ordinations to examine patterns among fish taxonomy and traits with geomorphology variables. Common geomorphology variables as predictors for taxonomic fish assemblages were river gradient, valley width, and valley slope. Significant geomorphology predictors of functional traits were valley width‐to‐floor width ratio, elevation, gradient, and channel sinuosity. These results provide evidence that fish assemblages respond similarly and strongly to geomorphic variables on two continents.

## Graphical abstract

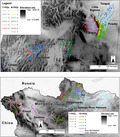

## 1 INTRODUCTION

Abiotic variables are successful predictors of taxonomic and functional diversity of fishes in river ecosystems (Griffith et al., 2005; Rahel & Hubert, 1991; Seelbach et al., 2006). For example, geomorphological variables such as elevation and channel slope are correlated with fish occurrences in high elevation mountain streams (Kruse et al., 1997; Lanka et al., 1987). River habitats used by fishes vary with quantity and structure of woody debris and with substrate size and distribution that are linked to valley and riparian characteristics (Grant & Swanson, 1995). Steep slopes of constrained valleys contribute increased dead trees, boulders, and alluvial fans, while wide valleys contribute fewer boulders and trees, and increased abundances of herbaceous plants and shrubs on riparian banks. In steep‐sided, narrow (constrained) valleys, riparian vegetation is similar to the adjacent hill slope (Grant & Swanson, 1995; Merrill et al., 2006). In contrast, in wide valleys, riparian species are adapted to smaller substrates and frequent inundation by floodwaters (Naiman & Decamps, 1997). Although these valley types and riparian zones are distinctive based on interactions with river ecosystems, they contribute substrate material and debris that are utilized by aquatic biota as habitat.

The riverine ecosystem synthesis (RES; Thorp et al., 2006; Thorp et al., 2021) predicts that biotic and abiotic stream characteristics including fish assemblages should vary with functional process zones (FPZ). The RES defines FPZs as distinct, patchy, and discontinuous river segments with unique valley and reach scale characteristics (Thorp et al., 2006). Sources of FPZ variation include precipitation, elevation, valley morphology, stream gradient, and sinuosity. The RES differs from the river continuum concept (RCC) because it posits that river ecosystem function should be driven by primarily patchy geomorphological variation, rather by a predictable and continuous upstream to downstream gradient (Vannote et al., 1980).

Evidence that fish assemblages respond to geomorphology variation has been observed at both local stream reach scales (Rieck & Sullivan, 2020) and larger valley scales (Elgueta et al., 2019; Maasri et al., 2021). Elgueta et al. (2019) found that dominant fish assemblages at catchment, valley, and channel scales were associated with distinct FPZs. Additionally, variation in valley‐scale geomorphology was a strong predictor of taxonomic fish assemblages for Mongolia forest steppe and grassland rivers (Maasri et al., 2021). Maasri et al. (2021) compared fish assemblage variation among reaches defined by geomorphology using occurrence‐ and abundance‐based beta diversities. Classification of fishes by functional traits frequently provides additional information about ecosystem roles in fish assemblage analyses (Pease et al., 2012). Robbins and Pyron (2021) identified three unique FPZs based on variation in channel width, floodplain width, and down valley slope for the mainstem of the Wabash River, USA. Significant differences in taxonomic and functional fish assemblages were found among FPZs and with river location (distance from confluence), supporting both the RCC and RES. However, variation of fish assemblages with hydrogeomorphology is largely unknown for forested and endorheic mountain streams of United States and Mongolian river ecosystems.

While mountain streams may represent rivers that inspired the RCC—rivers with montane headwaters that increase in volume with downstream distance (Vannote et al., 1980)—mountain rivers of endorheic regions likely have different functions. Endorheic rivers are confined to a drainage area and terminate in saline lakes (Great Salt Lake, Utah) or sink formations (Carson Sink, Nevada) (Benke & Cushing, 2011; Sigler & Sigler, 1987). Endorheic basin ecoregions are arid to semi‐arid drainages where rivers terminate in saline lakes or sink formations and eventually evaporate (Abell et al., 2008; Nichols, 2007). These ecoregions also tend to have low fish species richness in lotic habitats due to geographic isolation, but can have high endemism in lake and spring ecosystems (Probst et al., 2020; Sigler & Sigler, 1987). The limited dispersal ability of fishes to and from endorheic rivers, paired with low diversity and higher sensitivity to water withdrawal in desert ecoregions, further results in Great Basin fish assemblages that are highly sensitive to climate change (Jaeger et al., 2014).

The objectives of this study were to evaluate geomorphological variables as predictors of variation in mountain stream fish assemblages for forested and endorheic river ecosystems. Our hypothesis was that fish assemblages defined by taxonomy and functional traits will vary with geomorphology variables. We selected mountain watersheds in western United States and Mongolia to provide contrasts in human impacts. Western US rivers are impacted by dams, water diversions, agriculture management activities, and invasive species. Mountain rivers of Mongolia have few or no dams or water diversions or invasive species, and over‐grazing by livestock is the only agricultural impact (Altanbagana & Chuluun, 2010).

## 2 METHODS

### 2.1 Watershed and site selection

**FIGURE 1:**
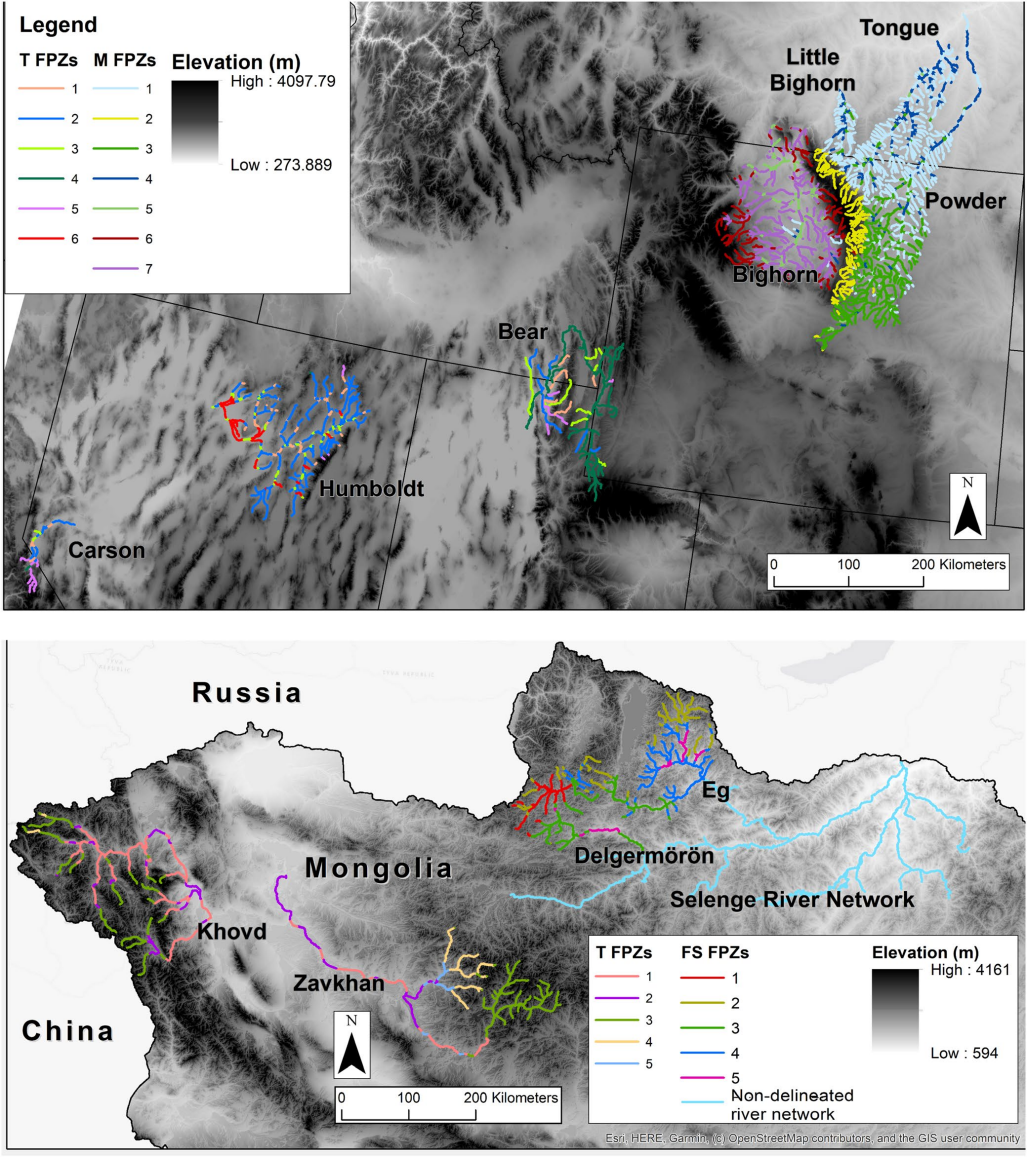
Rivers of the United States (top) and Mongolia (bottom) endorheic (T) and forested (FS) ecoregions

We used the ArcGIS‐based geomorphic model RESonate to identify distinctive reaches or FPZs separately in Mongolia and western North America mountain and endorheic ecosystems, following Williams et al. (2013) and Maasri et al. (2021). This is a GIS approach to identify unique river reaches using variables that are available in online GIS. We extracted geomorphic variables at 10‐km intervals, including the following: elevation, mean annual precipitation, geology, valley width, valley floor (floodplain) width, valley width‐to‐valley floor width ratio, river channel sinuosity, right valley slope, left valley slope, and down valley slope (Appendix 1). Data were normalized to a 0–1 scale, and a dissimilarity matrix was generated using a Gower dissimilarity transformation (Gower, 1971). The Gower transformation is recommended for nonbiological data when the measures are range‐standardized (Thoms et al., 2018). The dissimilarity matrix was used in a hierarchical clustering following the Ward linkage method, as it provided the best partitioning of cluster groups (Murtagh & Legendre, 2014). Additionally, we used a principal component analysis (PCA) to identify the contributive variables most important for group partitioning and to describe the cluster groups based on the ten variables identified above. Groups of 10‐km river sections, constituting discrete FPZs, were later mapped to allow for the identification of fish collection sites (Figure 1).

We performed the clustering using the cluster package (version 2.1.0; Maechler et al., 2018) and the PCA using the FactoMineR package (version 1.42; Lê et al., 2008) in R version 3.6.3 (Team R Core, 2020). We mapped the resulting groups using ArcGIS (version 10.5) (Figure 1).

### 2.2 Fish collections

We established reaches for fish collections measuring 20 times the mean wetted width of the river, calculated independently for each site. Fishes were collected by single‐pass backpack electrofishing with two netters (Ball State University IACUC #126193) following the American Fisheries Society standard collection protocols CPUE (Bonar et al., 2009). Electrofishing surveys were supplemented with seine netting and snorkel surveys when electrofishing was not possible due to low water conductivity for six headwater US Forested watershed sites. Seine netting resulted in fewer than one individual fish per site on average and was discontinued after collecting in US endorheic rivers. Snorkel surveys were performed in six headwater streams where water conductivity was too low for electrofishing. Snorkeling consisted of slowly swimming upstream for the site distance and recording all observed fishes. Fishes were identified to species, weighed to the nearest 0.01 g, measured in standard length (mm), and released. Fish species identifications and functional traits were from Mendsaikhan et al. (2017) for Mongolia or Poff and Allen (1995) for United States, and reproductive traits were from Balon (1975). Traits were substrate and stream size preferences, reproductive traits, and trophic traits.

### 2.3 Fish assemblage analyses

We evaluated fish assemblage responses to geomorphology using constrained ordinations with forward selection of environmental variables in CANOCO 5 software (canoco5.com). CANOCO evaluates length of the first ordination axis and recommends either a linear method (redundancy analysis, RDA) or a nonlinear method (canonical correspondence analysis, CCA). RDA is a direct gradient technique for multifactorial analysis of variance models using ecologically relevant distance measures and significance testing of individual variables (Legendre & Anderson, 1999). CCA is a direct gradient weighted averaging regression technique in CANOCO using environmental predictors (Palmer, 1993). Analyses were conducted for each ecoregion separately, and geomorphology data were reduced into fewer dimensions using principal components analysis (PCA) in Minitab version 18. Subsequent PCA axes were entered into RDA or CCA as environmental predictors of fish assemblage variation. If CANOCO was unable to reach a solution for constrained ordinations, we used unconstrained ordinations with environmental variables projected. CANOCO suggests either a linear PCA or unimodal (correspondence analysis) ordination based on gradient length. Fish and abundances by traits were log‐transformed by log (X + 1) before analysis to account for abundances spanning three orders of magnitude. We eliminated hybrid tiger trout from analyses as they were introduced and not capable of reproducing.

## 3 RESULTS

### 3.1 Geomorphological analysis

Functional process zones were classified using nine geomorphology variables and precipitation. Using the hierarchical method, four or five FPZs were defined for each river basin (Figure 1). The strongest contributing variables for FPZ delineation were valley width, elevation, and valley floor width. Geomorphology variables were reduced into PCA axes for each ecoregion and resulting PC axes by ecoregion were unique. US endorheic rivers were the Carson, Humboldt, and Bear rivers of the Great Basin ecoregion of the southwestern United States (*n* = 23 sites; Figure 1). US rivers were Tongue, Powder, and Ten Sleep rivers in the mountain ecoregions of the Yellowstone River watershed (Wyoming, US; *n* = 20 sites; Figure 1). Mongolian forested rivers were on the Eg River and Delgermurun rivers of north‐central Mongolia (*n *= 12 sites; Figure 1). Mongolia endorheic rivers were the Khovd and Zavkan rivers of western Mongolia (*n *= 13 sites; Figure 1). Site length varied from 40 to 1,500 m with a mean of 290 (*SD* = 266) m.

### 3.2 Fish assemblages

Fourteen of 19 species in the US endorheic ecoregion were invasive, and three of four species in the US forested ecoregion were invasive (Table 1). No invasive species were collected among the seven species from the Mongolia mountain ecoregion, or the five species from the Mongolia endorheic ecoregion (Table 1). Mean species richness (± *SD*) per site for the US endorheic ecoregion was 4.1 (± 2.1), 2.1 (± 0.8) for the US forested ecoregion, 2.7 (± 1.2) for the Mongolia endorheic ecoregion, and 3.9 (± 1.3) for the Mongolia forested ecoregion. Mean abundances (± SD) per site for the US endorheic ecoregion were 100 (± 113), 40 (± 29) for the US forested ecoregion, 97 (± 82) for the Mongolia forested ecoregion, and 59 (± 54) for the Mongolia endorheic ecoregion. We collected one endemic species in the US endorheic ecoregion (Tahoe sucker *Catostomus tahoensis*), one in the Mongolia endorheic ecoregion (Golubtsovi's stone loach, *Barbatula golubtsovi*), and one in the Mongolia forested ecoregion (Siberian dace, *Leuciscus leuciscus*).

**TABLE 1:** Fishes collected by country and ecoregion

Species	Common name	Country	Ecoregion	*n*
*Rhinichthys osculus*	Speckled dace	US	Endo	449
*Pimephales promelas*	Fathead minnow*	US	Endo	280
*Catostomus tahoensis*	Tahoe sucker	US	Endo	271
*Salvelinus fontinalis*	Brook trout*	US	Endo	227
*Cottus beldingii*	Paiute sculpin	US	Endo	220
*Lepomis cyanellus*	Green sunfish*	US	Endo	205
*Micropterus dolomieu*	Smallmouth bass*	US	Endo	169
*Cyprinus carpio*	Common carp*	US	Endo	140
*Salmo trutta*	Brown trout*	US	Endo	84
*Oncorhynchus mykiss*	Rainbow trout*	US	Endo	71
*Ameirus melas*	Black bullhead*	US	Endo	57
*Oncorhynchus clarki utah*	Bonneville cutthroat trout	US	Endo	43
*Gambusia affinis*	Mosquitofish*	US	Endo	23
*Catostomus platyrhynchus*	Mountain sucker	US	Endo	16
*Lepomis macrochirus*	Bluegill sunfish*	US	Endo	16
*Prosopium williamsoni*	Mountain whitefish	US	Endo	7
*Rhinichthys cataractae*	Longnose dace	US	Endo	3
*Richardsonius egregius*	Lahontan redside	US	Endo	3
*Micropterus salmoides*	Largemouth bass*	US	Endo	2
*Salmo trutta*	Brown trout*	US	For	215
*Salvelinus fontinalis*	Brook trout*	US	For	203
*Oncorhynchus clarki bouvieri*	Yellowstone cutthroat trout	US	For	107
*Oncorhynchus mykiss*	Rainbow trout*	US	For	46
*Barbatula barbatula*	Stone loach	Mongolia	Endo	460
*Thymallus brevirostris*	Mongolian grayling	Mongolia	Endo	110
*Barbatula conilobus*	Conilobus’ stone loach	Mongolia	Endo	109
*Oreoleuciscus potanini*	Altai osman	Mongolia	Endo	89
*Barbatula golubtsovi*	Golubtsovi's stone loach	Mongolia	Endo	2
*Phoxinus phoxinus*	Common minnow	Mongolia	For	845
*Brachymystax lenok*	Lenok	Mongolia	For	98
*Barbatula barbatula*	Stone loach	Mongolia	For	85
*Thymallus arcticus*	Arctic grayling	Mongolia	For	34
*Leuciscus leuciscus*	Common dace	Mongolia	For	13
*Esox lucius*	Northern pike	Mongolia	For	4
*Perca fluviatilis*	European perch	Mongolia	For	1

Note

Endo designates the endorheic basins and for the forested basins. Asterisks * refer to invasive species.

### 3.3 US forested ecoregion

**FIGURE 2:**
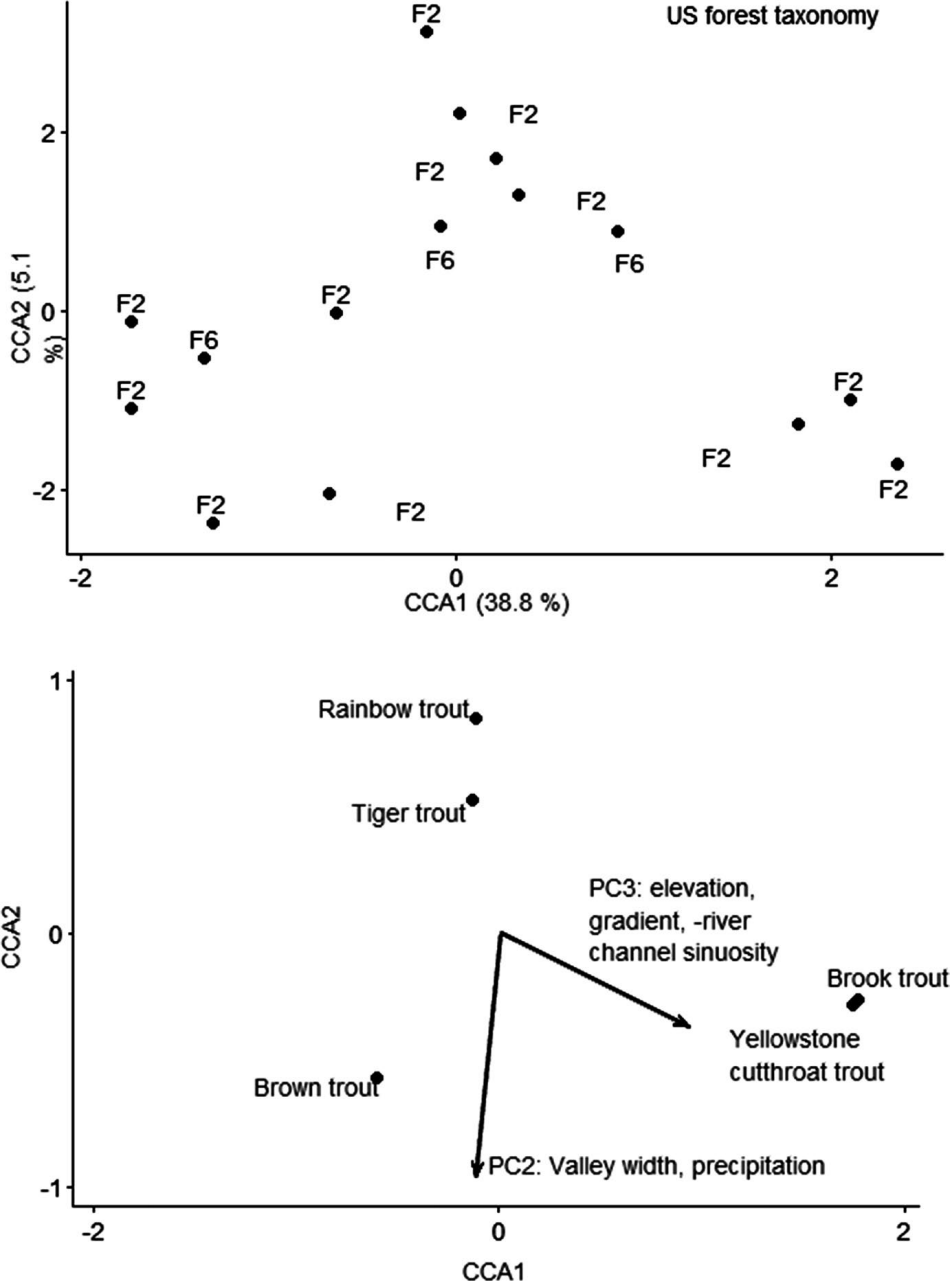
Canonical correspondence analysis ordination of fishes by taxonomy for US forested ecoregion. Top figure is sites; bottom figure is species and vectors for the significant predictor. Sites are labeled by functional process zone

CANOCO recommended a unimodal ordination (CCA) based on gradient of 3.3 SD units for the US forested taxonomy data. The CCA ordination for the US forested ecoregion using taxonomy resulted in two axes that explained 35.2% and 31.1% of variation (Figure 2). The forward selection procedure in the CCA resulted in only geomorphology PC3 as significant; this PC axis was driven by river channel sinuosity, elevation, and gradient (Table 2). The CCA first axis was significant (*F* = 2.3, *p* = .022), and all CCA axes together were significant (*F* = 4.1, *p* = .008; Table 2). Brook trout and Yellowstone cutthroat trout occurred at higher elevations, with decreased channel sinuosity compared to rainbow trout and tiger trout. Brown trout tended to occur in streams with wider valleys and increased annual precipitation.

**TABLE 2:** Results for constrained ordination analyses of fishes by ecoregion, by taxonomy and functional traits

Ecoregion	Contribution %	Pseudo‐*F*	*p* (adj)
US forested taxonomy			
PC3: elevation, gradient, − river channel sinuosity	66.5	7.1	.018
PC2: valley width, precipitation	25.9	4.1	.021
US endorheic taxonomy			
PC1: − gradient, + left valley slope, valley width‐to‐floor width ratio	47.7	2.8	.006
Mongolia endorheic traits			
PC2: valley width‐to‐floor width ratio, gradient, precipitation	51.8	5.4	.048
PC3: − channel sinuosity, − valley width, left valley slope	44.4	7.3	.018

**FIGURE 3:**
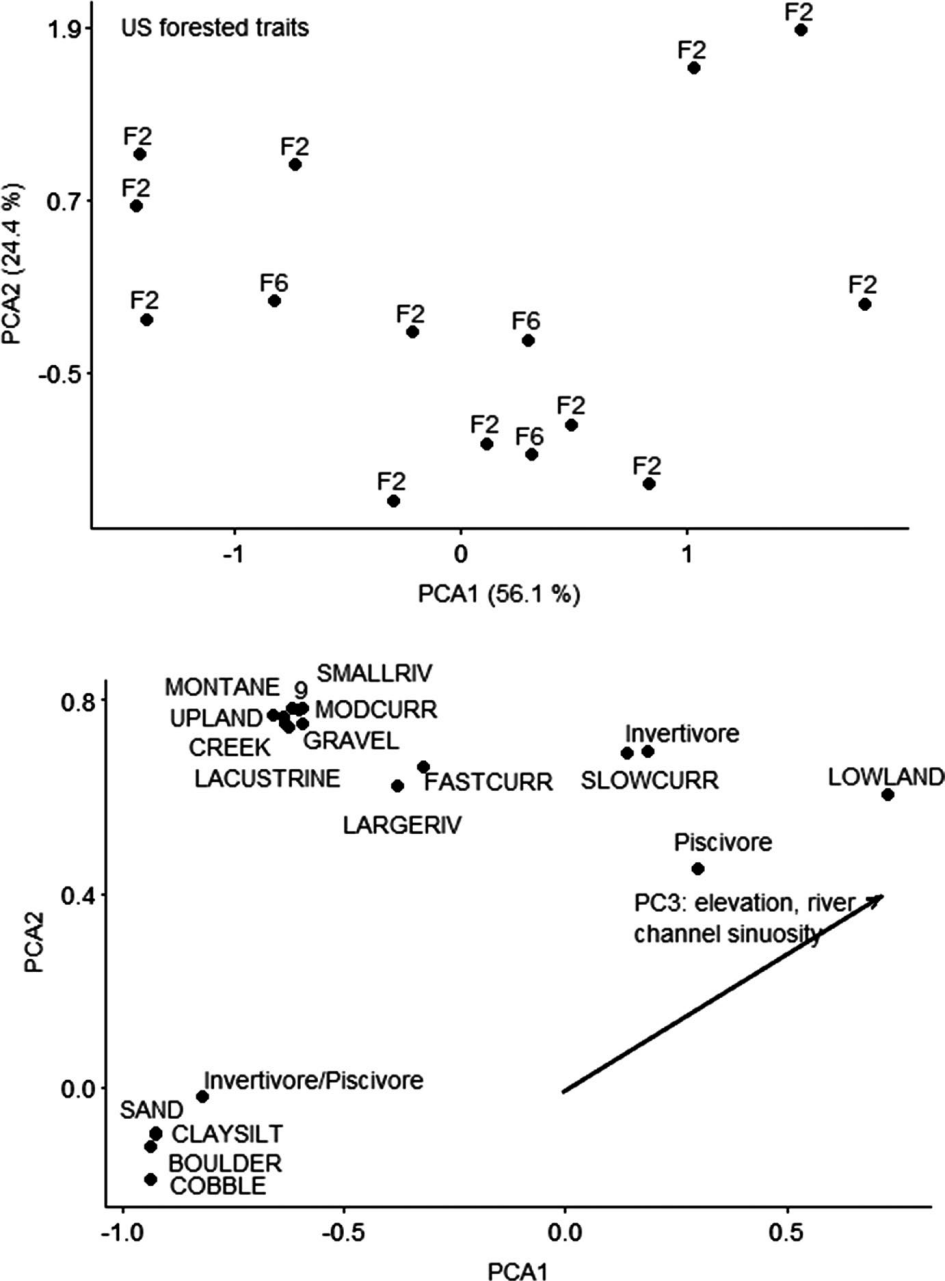
Principal components analysis ordination of fishes by traits for US forested ecoregion. Top figure is sites; bottom figure is species and vectors for the significant predictor. Sites are labeled by functional process zone. Trait numbers refer to reproduction mode

CANOCO recommended a linear ordination (RDA) based on gradient of 1.1 SD units for the US forested trait data. The RDA ordination for the US forested ecoregion using traits did not result in a significant result. The unconstrained ordination selected was PCA with projection of environmental variables, because the gradient was 1.0 SD long. The first PCA axis explained 56.1% of variation, and the second PCA axis explained 24.4% of variation. Variation explained by the environmental variables was 17.0% on the first axis and 7.1% on the second axis, and PC3 was strongly correlated with the first and second axis (Figure 3). PC3 explained elevation and river channel sinuosity variation, with positive loadings for sites with higher elevation and increased sinuosity. PC1 explained valley floor width with negative loadings for sites with lower valley floor widths. Fishes that are lithophils (reproductive mode 9, bury their eggs in gravel) that are piscivores with preferences for montane streams with all substrates except for sand, clay/silt, boulder, and cobble, occurred at sites with higher elevation and increased channel sinuosity.

### 3.4 US endorheic ecoregion

**FIGURE 4:**
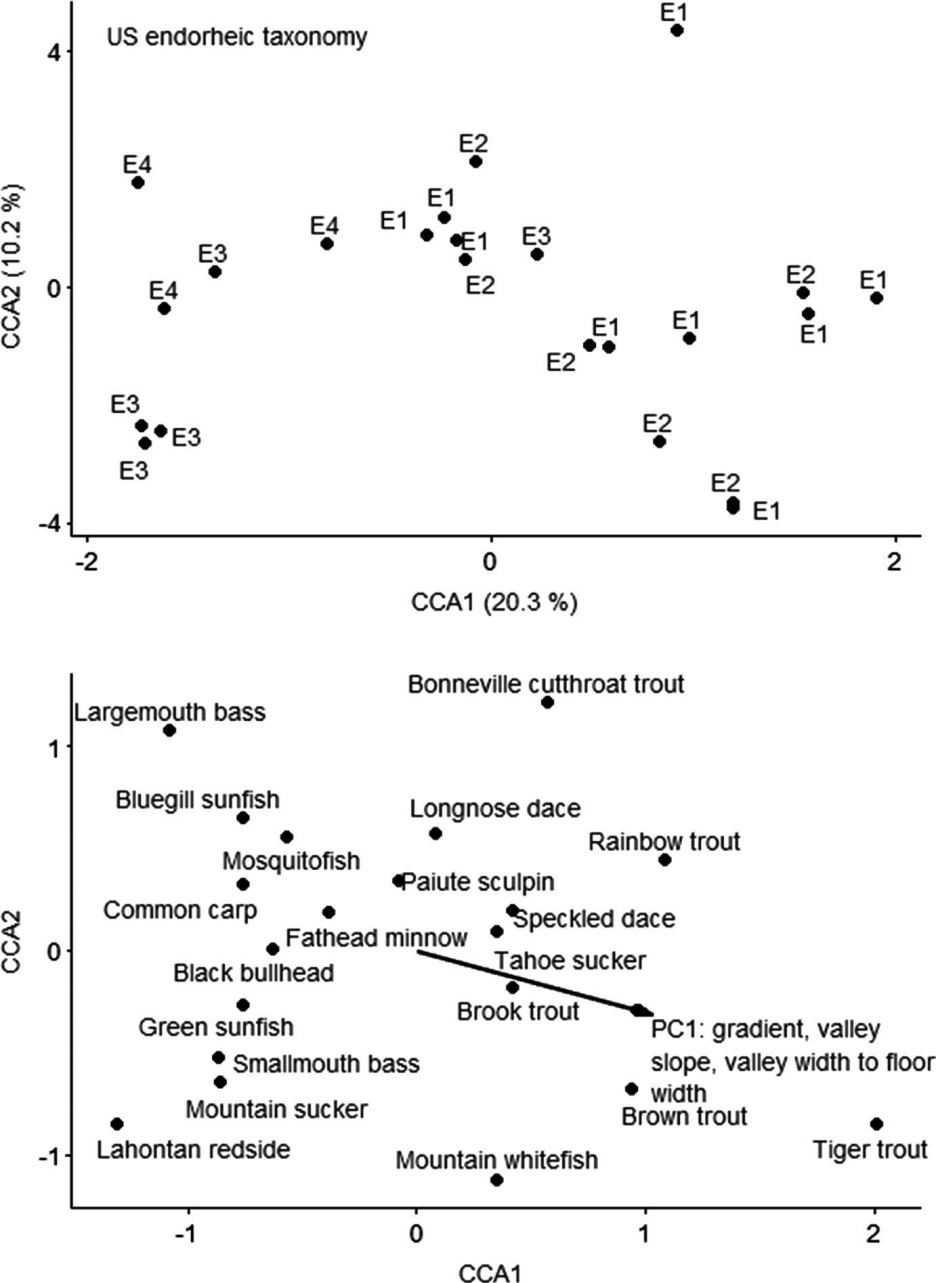
Canonical correspondence analysis ordination of fishes by taxonomy for US endorheic ecoregion. Top figure is sites; bottom figure is species and vectors for the significant predictor. Sites are labeled by functional process zone

Response data resulted in a gradient of 7.8 SD units; thus, we used a nonlinear ordination (CCA). The CCA ordination for the US endorheic ecoregion using taxonomy resulted in first two axes that explained 10.2% and 20.1% of variation (Figure 4). The forward selection procedure resulted in geomorphology PC1 as a significant predictor. PC1 represents gradient, left valley slope, and valley width‐to‐floor width ratio. The CCA first axis was not significant, but all axes together were significant (*F* = 1.7, *p* = .014). Sites with higher gradients, higher valley slope, and higher valley width‐to‐floor width ratio had higher abundances of brown trout and tiger trout, and lower abundances of other species.

**FIGURE 5:**
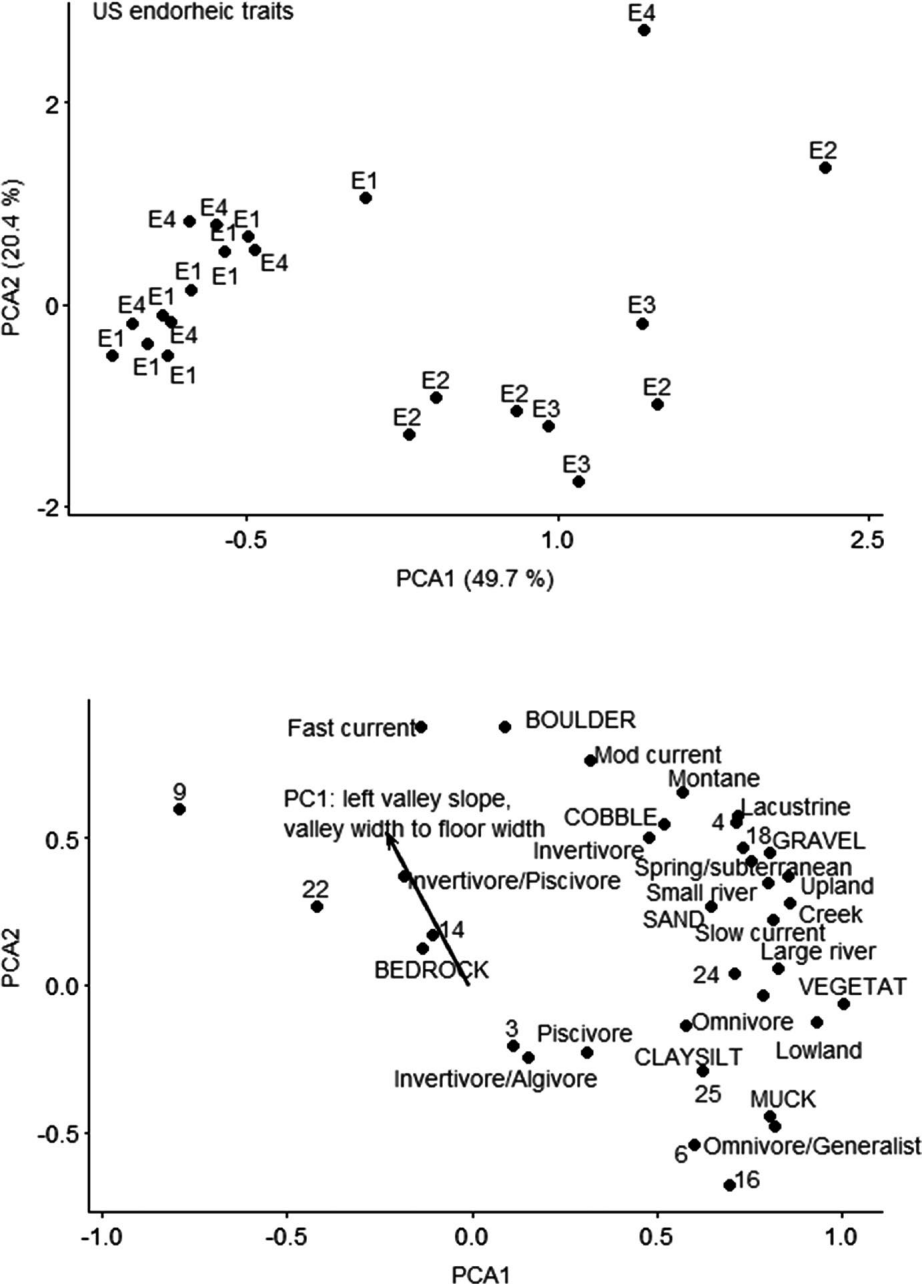
Principal components analysis ordination of fishes by traits for US endorheic ecoregion. Top figure is sites; bottom figure is species and vectors for the significant predictor. Sites are labeled by functional process zone. Trait numbers refer to reproduction mode

CANOCO recommended a linear method (RDA) based on gradient of 1.5 SD units for the US endorheic traits data. None of the predictor variables were significant in the RDA ordination for US endorheic ecoregion using traits. The unconstrained ordination selected was PCA with projection of environmental variables, because the gradient was 1.4 SD long. The first PCA axis explained 49.7% of variation, and the second PCA axis explained 20.4% of variation. Variation explained by the environmental variables was 4.5% on the first axis and 10.7% on the second axis, and PC1 was strongly correlated with the first and second axes (Figure 5). PC1 explained left valley slope and valley width‐to‐floor width ratio, with positive loadings for sites with higher slopes and higher valley width‐to‐floor width ratios. Sites with higher valley slope and increased valley width‐to‐floor width ratios had fishes with reproductive modes of lithophils (reproductive mode 9, bury their eggs in gravel) or nest spawners (reproductive mode 22, build structures for egg deposition) that were invertivore/piscivores and prefer streams with fast current and boulder substrates.

### 3.5 Mongolia endorheic ecoregion

**FIGURE 6:**
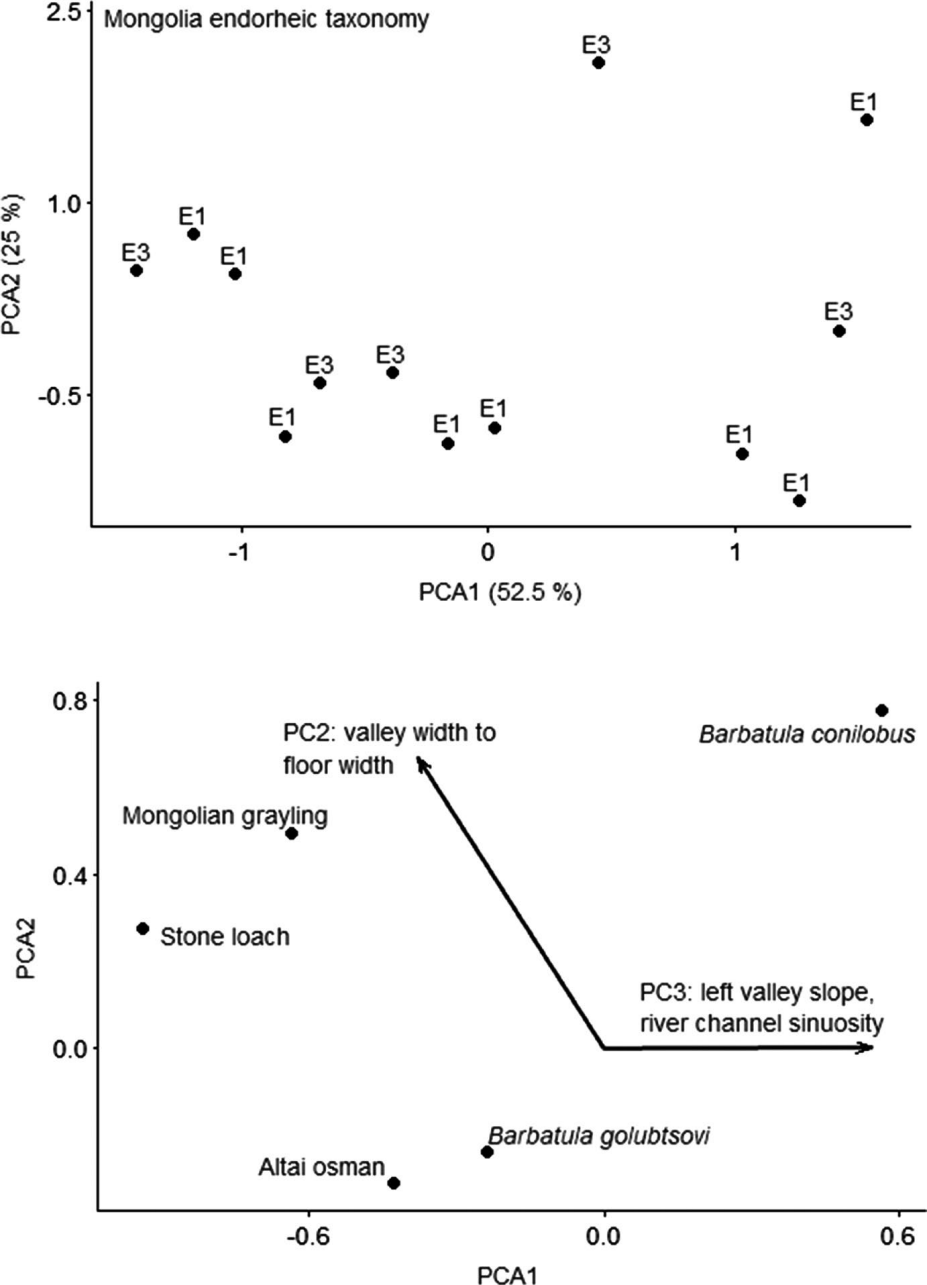
Principal components analysis ordination of fishes by taxonomy for Mongolia endorheic ecoregion. Top figure is sites; bottom figure is species and vectors for the significant predictor. Sites are labeled by functional process zone

CANOCO recommended a linear method (RDA) based on gradient of 2.3 SD units for the Mongolia endorheic taxonomy data. None of the predictors for the RDA ordination for the Mongolia endorheic ecoregion fish by taxonomy were significant. The unconstrained ordination selected was PCA with projection of environmental variables, because the gradient was 1.8 SD long. The first PCA axis explained 52.5% of variation, and the second PCA axis explained 25% of variation. Variation explained by the environmental variables was 18.8% on the first axis and 15.6% on the second axis. PC2 was strongly correlated with the first and second axes, and PC3 was correlated with the first axis (Figure 6). PC2 explained valley width‐to‐floor width ratio, with positive loadings for sites with higher valley width‐to‐floor width ratios. PC3 explained left valley slope and river channel sinuosity, with positive loadings for sites with higher slope and decreased sinuosity. Mongolia grayling occurred at sites with higher valley width‐to‐floor width ratios compared to other species. *Barbatula conilobus* loaches occurred at sites with higher valley slope and increased channel sinuosity compared to other species.

**FIGURE 7:**
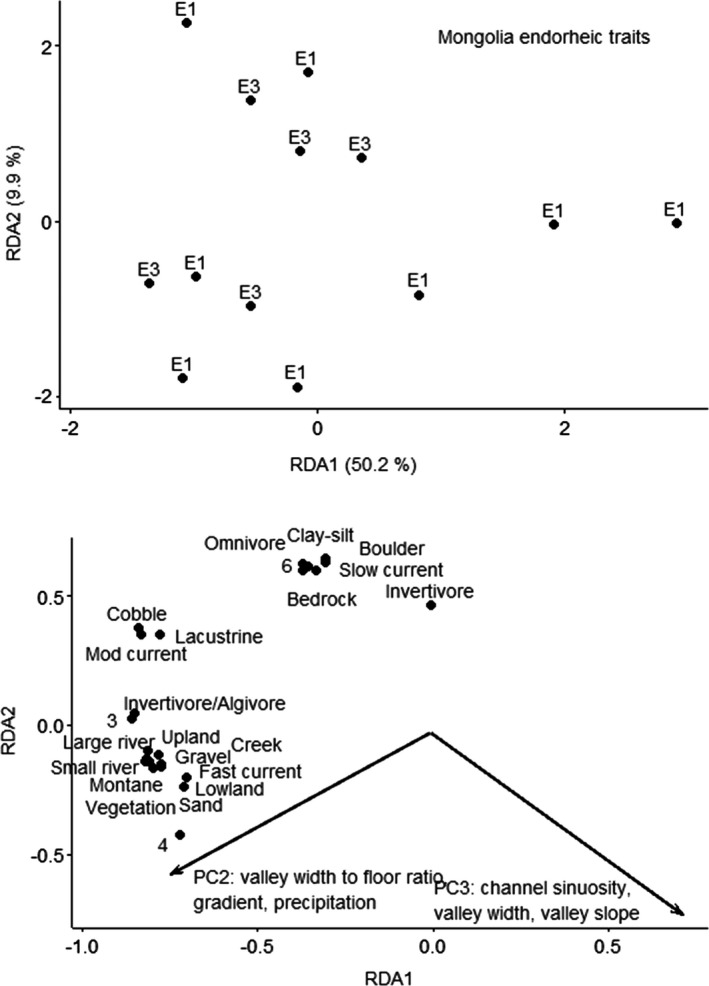
Ordination (RDA) of fishes by traits for Mongolia endorheic ecoregion. Top figure is sites; bottom figure is species and vectors for the significant predictor. Sites are labeled by functional process zone and trait numbers refer to reproduction mode

CANOCO recommended a linear method (RDA) based on gradient of 1.2 SD units for the Mongolia endorheic traits data. The RDA ordination for the Mongolia endorheic ecoregion using traits resulted in two axes that explained 50.2% and 11% of variation (Figure 7). The forward selection procedure resulted in PC2 and PC3 as significant. PC2 was driven by valley width‐to‐floor ratio, stream gradient, and precipitation, and PC3 was driven by channel sinuosity, valley width, and valley slope. The RDA first axis was significant (*F* = 3.0, *p* = .014), and all RDA axes were significant (*F* = 5.2, *p* = .006). Fishes with reproductive modes of pelagolithophils (reproductive mode 3, deposit eggs on rocks or gravel bottom, in some larvae become buoyant) or lithophils (reproductive mode 4, deposit eggs rock or gravel) that were invertivore/algivores and prefer vegetation, gravel, and sand substrates and multiple current velocities and stream sizes occurred at sites with higher valley width‐to‐valley floor ratios, higher gradients, and increased annual precipitation. Fishes with reproductive mode of phytolithophils (reproductive mode 6, deposit eggs in clear water on plants or other submerged items) that were omnivores or invertivores, and prefer boulder and bedrock substrates with silt, and slow current occurred at sites with decreased channel sinuosity and decreased valley slope.

### 3.6 Mongolia forested ecoregion

**FIGURE 8:**
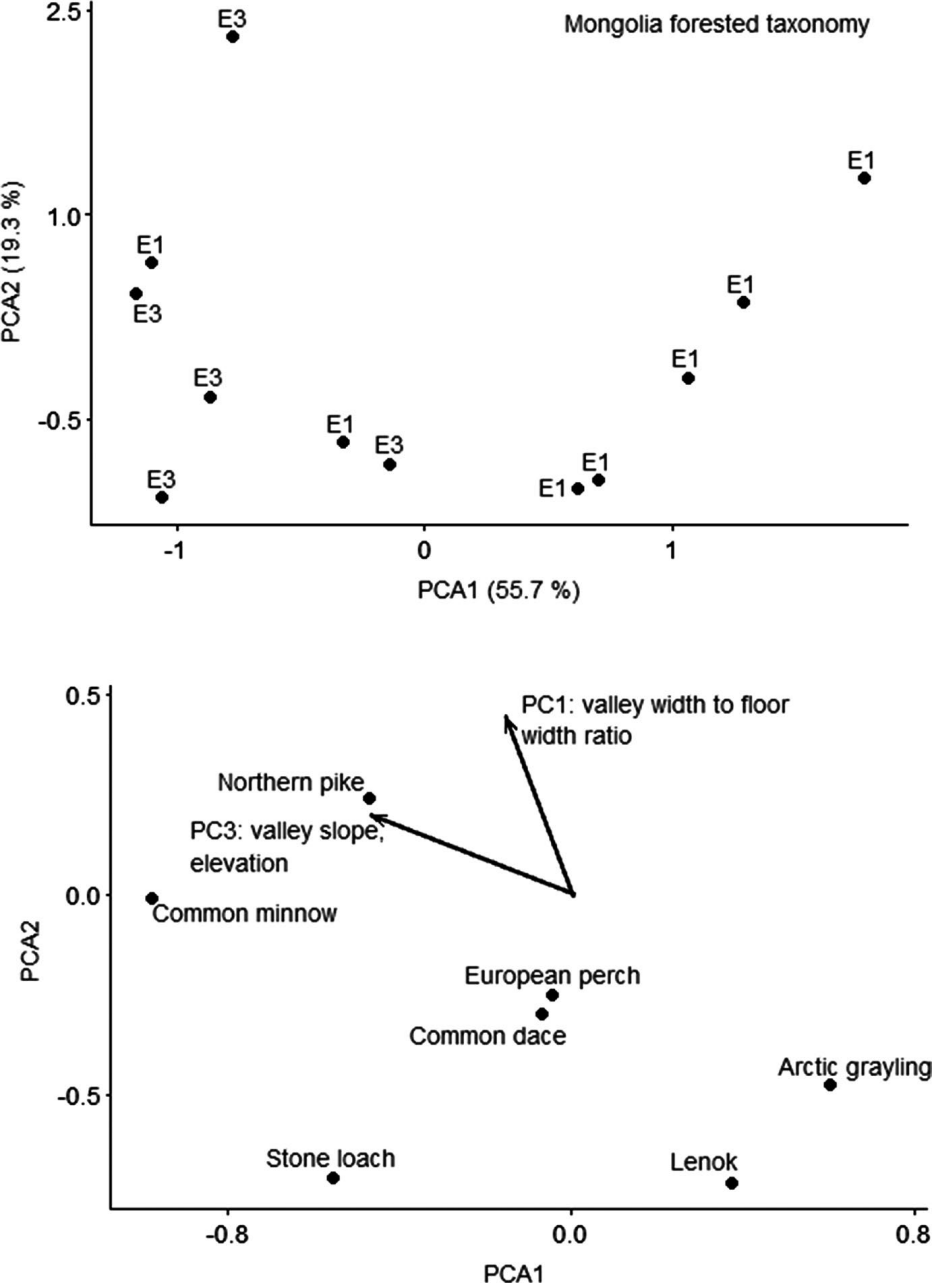
Principal components analysis ordination of fishes by taxonomy for Mongolia forested ecoregion. Top figure is sites; bottom figure is species and vectors for the significant predictor. Sites are labeled by functional process zone

CANOCO recommended a linear method (RDA) based on gradient of 2.1 SD units for the Mongolia forested taxonomy data. CANOCO recommended a linear method (RDA) based on gradient of 1.9 SD units for the Mongolia forested traits data. Neither of the RDA ordinations for the Mongolia forested ecoregion fish by taxonomy or traits resulted in significant predictors. The unconstrained ordination selected for taxonomy data was PCA with projection of environmental variables, because the gradient was 1.8 SD long. The first PCA axis explained 55.7% of variation, and the second PCA axis explained 19.3% of variation. Variation explained by the environmental variables was 8.2% on the first axis and 6.5% on the second axis. PC1 was strongly correlated with the second axis, and PC3 was correlated with the first and second axes (Figure 8). PC1 explained valley width‐to‐floor width ratio, with positive loadings for sites with lower valley width‐to‐floor width ratios. PC3 explained right valley slope and elevation, with positive loadings for sites with higher slope and lower elevation. Sites with higher elevation, higher slopes, and higher valley width‐to‐valley floor width ratios had higher abundances of northern pike compared to other species.

**FIGURE 9:**
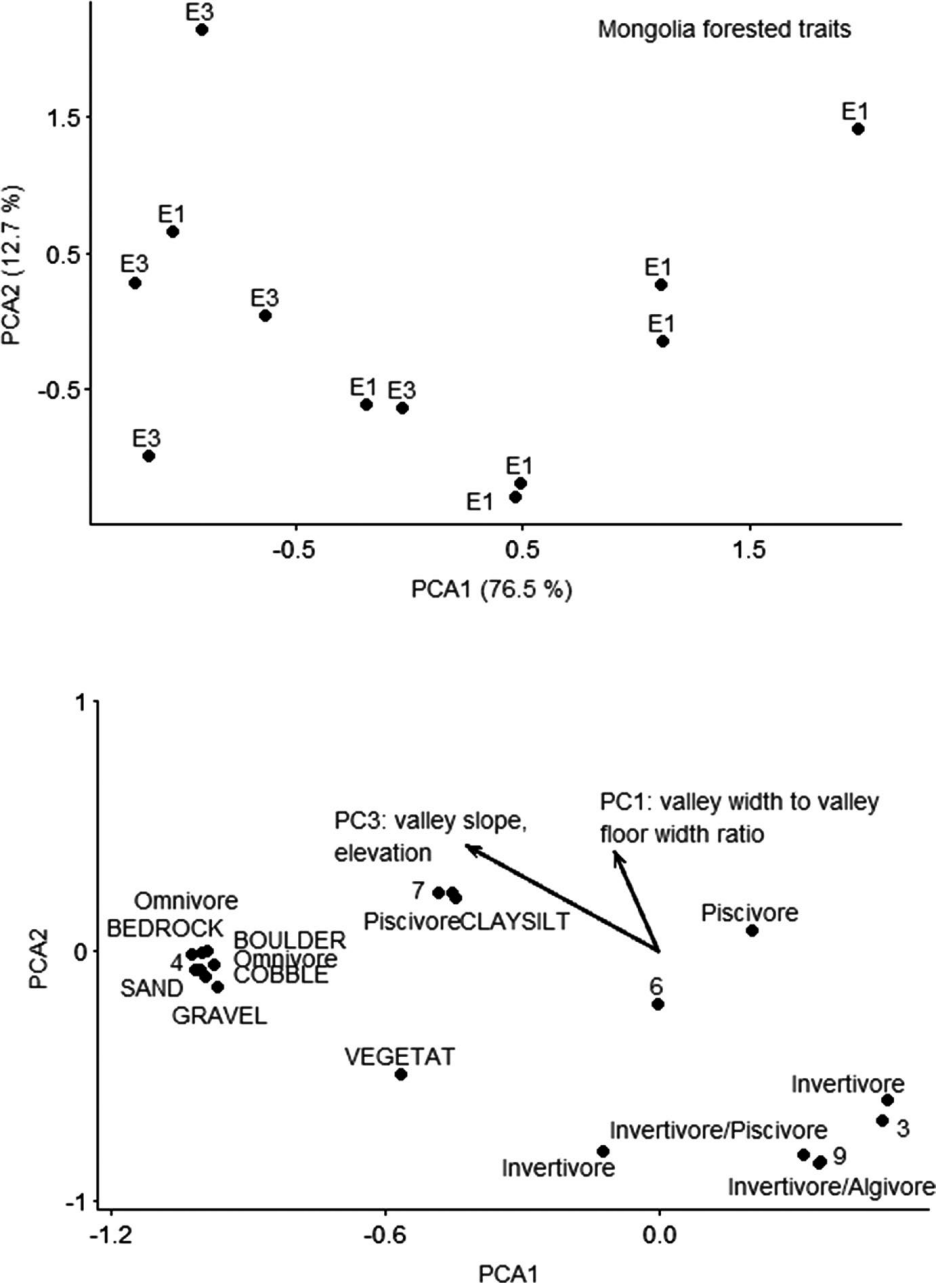
Principal components analysis ordination of fishes by traits for Mongolia endorheic ecoregion. Top figure is sites; bottom figure is species and vectors for the significant predictor. Sites are labeled by functional process zone and trait numbers refer to reproduction mode

The unconstrained ordination selected for trait data was PCA with projection of environmental variables, because the gradient was 1.7 SD long. The first PCA axis explained 76.5% of variation, and the second PCA axis explained 12.7% of variation. Variation explained by the environmental variables was 7.7% on the first axis and 9.1% on the second axis. PC1 was strongly correlated with the first axis, and PC3 was correlated with the first and second axes (Figure 9). PC1 explained valley width‐to‐floor width ratio, with positive loadings for sites with lower valley width‐to‐floor width ratios. PC3 explained right valley slope and elevation, with positive loadings for sites with higher slope and lower elevation. Fishes with reproductive modes of phytophils (reproductive mode 7, scatter eggs with an adhesive membrane that sticks to plant or logs) or lithophils (reproductive mode 4, deposit eggs rock or gravel) that were piscivores or omnivores that prefer multiple substrates had higher abundances at sites with increased valley slopes and increased valley width‐to‐valley floor width ratios.

## 4 DISCUSSION

### 4.1 Effects of geomorphology on fish assemblage structure

Our results showed that geomorphology has a strong influence on fish assemblages in US forested and endorheic rivers and in Mongolia endorheic rivers, confirming recent results showing how changes in valley‐scale geomorphology drives changes in fish assemblages in river macrosystems (Maasri et al., 2021). Geomorphic variables that were important in predicting fish occurrences tended to distinguish reaches based on valley width and gradient. However, the variables that were significant varied among ecoregions and whether we used fishes classified by taxonomy or by functional traits. Our interpretation is that depauperate fish assemblages of ecoregions resulted in low ability to explain variation using geomorphology.

At fine scales, habitat type, complexity, and hydrological regime dictate the presence of many stream organisms. However, at coarse scales, riparian vegetation, nutrient loading, and establishment of nonindigenous species contribute the largest effect on fish assemblage variation (Hermoso et al., 2011; Schlosser, 1991). However, anthropogenic alteration of river habitats, including drainage of wetlands and forests for row crop agriculture, channelization, and snag removal for the facilitation of barge traffic, and the erection of impoundments for flood control and retention of usable water have widespread effects on natural community assemblages (Nilsson et al., 2005). Fishes in high elevation mountain stream ecosystems are currently restricted by fewer anthropogenic variables than fishes in lower elevation rivers (e.g., upstream of dams and nonpoint source pollutants). However, historic alterations such as beaver removal and mining in the mountain steppes of the western United States resulted in incision of stream channels and contamination by heavy metals (Wohl, 2006). The majority of fishes in this region are stocked or re‐introduced by state agencies, impairing the biotic integrity of local ecosystems. Conversely, in Mongolia, forested steppe rivers are not altered through large impoundments or stocking of native or non‐native species, but they are impacted by widespread and intensive livestock grazing and mining activities (Chalov et al., 2012). Future alterations to Mongolian rivers are currently in planning stages and include the installation of high‐head dams and introductions of exotic salmonid and centrarchid species for recreational fishing (Simonov et al., 2017).

Our results suggest that valley width and gradient are effective predictors of assemblage variation and species occurrence in mountain rivers. Riparian zones of rivers link valley width to river ecosystem characteristics and are strongly influenced by valley characters (Merrill et al., 2006). Riparian contributions to physical stream features include additions of organic material, woody debris, and indirectly control of nutrient inputs (Gregory et al., 1991). Valley geomorphology influences the composition of substrates and the way in which material is deposited in the river. The width of a valley can influence the rate at which this material is deposited into lotic ecosystems (Leopold et al., 1964), resulting in distinctive fish habitat features. Walters et al. (2003) found this for Piedmont streams when they compared fish assemblages among sites with different reach characteristics.

Fish assemblage variation in constrained and wide valleys can result through four possible mechanisms. First, substrate variation may strongly influence fish assemblages (Mueller & Pyron, 2010); constrained valley rivers generally have higher velocity water and larger substrates than wide valley rivers. These larger substrates in constrained valleys contribute to the availability of a second potential mechanism contributing to the organization of fish assemblages: fish habitat and cover (Stefferud et al., 2011). Constrained valley rivers substrates tend toward large cobble, boulders, and logjams that are sourced from the valley slope and provide important for fish cover, whereas wide valley rivers frequently contain increased riparian vegetation and macrophytes likely due to overall lower water velocities and decreased abundance of large woody debris due to their gradually sloping banks. A third mechanistic category for valley width control on fish assemblages is food web variation (Smits et al., 2015). Less direct sunlight reaches rivers in constrained valley rivers compared to wide valley rivers. This is expected to result in lower primary productivity and variation in basal carbon sources. A fourth mechanistic category for valley width control on fish assemblages is competitive interaction among fish species. Survival and recruitment of smaller forage fishes varies in the presence of dominant predators or available resources in a stream reach. We suggest that all four of these mechanisms contribute to the observed variation in fish assemblages mediated by valley width.

The US Great Basin contains several species of endemic pupfishes and suckers, as well as the Lahontan and Bonneville subspecies of cutthroat trouts (*Onchorhynchus clarki*). Multiple fish introductions and experimental fisheries near the end of the 19th and first part of the 20th century occurred in this ecoregion, starting with overfishing of native species, then introductions of numerous invasive species including common carp (*Cyprinus carpio*), smallmouth bass (*Micropterus dolomieu*), brown trout (*Salmo trutta*), and black bullhead (*Ameiurus melas*) (Sigler & Sigler, 1987). To further evaluate the validity of these valley width associations, we compared published habitat preferences of species in Table 1 to habitat variables associated with valley type from our collections. Cunjak and Green (1983) found that rainbow trout (*Oncorhynchus mykiss)* inhabit higher velocity streams than brook trout. Brown trout are described as a habitat generalist (Ayllón et al., 2010) although Vismara et al. (2001) found that adult brown trout in Italian rivers preferred habitats with large boulders and overhanging vegetation. Although we found the highest abundances of rainbow trout and brook trout in constrained valleys, our observations during snorkel surveys showed these taxa inhabit different microhabitats. Speckled dace (*Rhinichthys osculus)* inhabit faster moving waters than other desert species (Scoppettone et al., 2005) and adults utilize large stones as cover (Peden & Hughes, 1981). We found the highest abundances of speckled dace in constrained valleys with high water velocity. Adult Tahoe sucker prefer large substrates (Moyle & Vondracek, 1985), which is consistent with constrained valley habitats where we found them. Adult cutthroat trout habitat is characterized by overhanging vegetation and availability of pool habitat (Kruse et al., 2000), similar to habitats in wide valleys where we collected them. Other native species occurred in wide valley habitats as predicted: common minnow prefer relatively shallow, slow‐moving lotic habitats but do not have preferences for substrate size (Lamouroux et al., 1999). Mountain sucker (*Catostomus platyrhynchus)* inhabit riffles and fast water habitats with substrates ranging from sand to boulders (Sigler & Sigler, 1987). Detailed valley type habitat preferences are not available for Mongolia fishes.

The invasive species we collected did not occur in predicted patterns for constrained vs. wide valley habitats. Fathead minnow are generalists and are associated with pool and backwater habitats (Quist et al., 2005). Black bullhead are considered effective invasive species due to their status as habitat generalists for feeding and reproduction (Novomeská & Kováč, 2009), although they prefer aquatic vegetation cover (Stuber, 1982). Western mosquitofish (*Gambusia affinis*) prefer calm water over fast‐moving rivers (Casterlin & Reynolds, 1977) and inhabit a wide range of habitats (Pyke, 2005). Three invasive species occurred in habitats as predicted for wide valleys. Common carp occur in slow‐moving waters with small substrates (Butler & Wahl, 2010. Smallmouth bass and green sunfish are invasives that are strongly associated with large substrates, boulders, large woody debris, and root wads in slow‐moving stream habitats (Stuber et al., 1982; Todd & Rabeni, 1989).

Each of these habitat and hydrology preferences are concordant with habitat variation that is expected to characterize valley types. Species that prefer faster water and large substrates frequently occur in constrained valley reaches, while species that prefer slower flows or algae and macrophyte growth occur in wide valley reaches. These results may not be applicable across ecoregions or river drainages, but our method of river classification can be implemented in any lotic habitat requiring decisions for stocking success or conservation of fish species. This study demonstrates the utility of spatial analysis in predicting functional process zones and fish assemblage variation in endorheic basins and forest steppe ecoregions of two continents. Future research should evaluate associations of habitat type with valley width and fish preferences for habitat in each ecoregion.

## CONFLICT OF INTEREST

None declared.

## AUTHOR CONTRIBUTIONS

**Robert Shields:** Conceptualization (lead); Data curation (equal); Formal analysis (lead); Investigation (equal); Methodology (equal); Writing‐original draft (lead). **Mark Pyron:** Conceptualization (equal); Data curation (lead); Funding acquisition (equal); Investigation (lead); Methodology (equal); Project administration (equal); Writing‐original draft (equal). **Emily R. Arsenault:** Conceptualization (supporting); Data curation (equal); Investigation (equal); Methodology (equal); Writing‐review & editing (equal). **James H. Thorp:** Conceptualization (equal); Funding acquisition (lead); Methodology (equal); Writing‐review & editing (equal). **Mario Minder:** Investigation (equal); Writing‐review & editing (equal). **Caleb Artz:** Investigation (supporting); Writing‐review & editing (equal). **John Costello:** Investigation (supporting); Writing‐review & editing (equal). **Amarbat Otgonganbat:** Investigation (supporting); Writing‐review & editing (equal). **Bud Mendsaikhan:** Investigation (supporting); Writing‐review & editing (equal). **Solongo Altangerel:** Investigation (supporting). **Alain Maasri:** Conceptualization (equal); Funding acquisition (equal); Investigation (equal); Writing‐review & editing (equal).

## Data Availability

Fish abundance and geomorphology PCA data are available on the Dryad Digital Repository https://doi.org/10.5061/dryad.9s4mw6mhr.

## APPENDIX 1

Sites with Functional Process Zones and elevation. Site ID refers to river acronym and FPZ acronym

**Table:**

Cont/Basin	Site ID	Latitude	Longitude	Elevation
USA Terminal	BEAUC1	42.13735	−111.643	1362.260
USA Terminal	BEAUC2	40.87426	−110.836	2553.515
USA Terminal	BEAUC3	40.88673	−110.798	2411.870
USA Terminal	BEAUW1	42.52684	−111.578	767.930
USA Terminal	BEAUW2	41.60423	−111.587	1610.249
USA Terminal	BEAUW3	40.92834	−110.736	2632.322
USA Terminal	CARLC1	39.10872	−119.712	1409.978
USA Terminal	CARLC2	39.13975	−119.704	1395.571
USA Terminal	CARLC3	39.17526	−119.682	1357.501
USA Terminal	CARLW1	39.24841	−119.584	1317.421
USA Terminal	CARLW2	39.2857	−119.417	1294.629
USA Terminal	CARLW3	39.28925	−119.288	1276.173
USA Terminal	CARUC1	38.718	−119.922	2170.898
USA Terminal	CARUC2	38.77618	−119.896	1966.324
USA Terminal	CARUC3	38.57726	−119.697	1851.015
USA Terminal	CARUW1	38.68522	−119.93	2170.898
USA Terminal	CARUW2	38.75284	−119.936	1966.324
USA Terminal	CARUW3	38.58747	−119.688	1851.015
USA Mountain	CLEUC3	44.27635	−106.953	2104.350
USA Mountain	CRAUW1	44.16916	−106.915	1933.386
USA Mountain	CRAUW2	44.19484	−106.928	1933.386
MNG Mountain	DELLW1	49.62554	99.57769	1322.000
MNG Mountain	DELLW2	49.62298	99.68978	1309.000
MNG Mountain	DELLW3	49.63669	99.93284	1282.000
MNG Mountain	DELUC1	50.17475	98.48839	1319.000
MNG Mountain	DELUC2	50.17598	98.48741	1319.000
MNG Mountain	DELUC3	50.10378	98.60467	1650.072
MNG Mountain	DELUC4	50.12805	98.63965	1650.072
MNG Mountain	EGILS1	50.5221	101.438	1125.249
MNG Mountain	EGILS2	50.50471	101.7512	1096.000
MNG Mountain	EGILS3	50.09561	101.5926	1140.000
MNG Mountain	EGILS4	50.31176	101.941	1059.000
MNG Mountain	EGILW1	50.56906	101.529	1120.099
USA Terminal	HUMLC1	40.72666	−116.01	1501.671
USA Terminal	HUMLC2	40.5791	−116.277	1461.810
USA Terminal	HUMUC1	40.66423	−115.448	1950.201
USA Terminal	HUMUC2	40.65824	−115.433	1950.201
USA Terminal	HUMUC3	40.68977	−115.477	1814.967
MNG Terminal	KVDUC1	48.87218	89.68016	1755.000
MNG Terminal	KVDUC2	48.83362	89.53129	1769.000
MNG Terminal	KVDUC3	48.76865	90.16923	2040.716
MNG Terminal	KVDUW1	49.18522	89.20849	1816.635
MNG Terminal	KVDUW2	48.89527	89.64735	1755.000
MNG Terminal	KVDUW4	48.86972	90.1707	1858.000
USA Mountain	LAKUW1	44.19248	−107.21	2374.238
USA Mountain	LBHUW1	44.79442	−107.689	2453.036
USA Mountain	LBHUW2	44.80872	−107.721	2579.264
USA Mountain	LBHUW3	44.79861	−107.765	2470.364
USA Mountain	SOUUW1	44.25207	−106.953	2246.415
USA Mountain	TENUC2	44.23128	−107.232	2457.672
USA Mountain	TENUW1	44.24383	−107.222	2706.522
USA Mountain	TENUW2	44.20452	−107.237	2457.672
USA Mountain	TONUC1	44.72168	−107.449	2358.568
USA Mountain	TONUC2	44.68661	−107.446	2554.787
USA Mountain	TONUC3	44.77088	−107.471	2143.423
MNG Terminal	ZAKLC1	48.27912	93.48143	1123.533
MNG Terminal	ZAKLW1	48.3215	93.4944	1110.000
MNG Terminal	ZAKUC1	47.17798	97.721	2137.000
MNG Terminal	ZAKUC2	47.09143	97.63524	2116.000
MNG Terminal	ZAKUC3	47.03971	97.60476	2086.000
MNG Terminal	ZAKUC4	47.27719	98.05714	2247.000
MNG Terminal	ZAKUC5	46.58155	97.25419	1782.481
MNG Terminal	ZAKUW1	47.22467	97.6156	2143.000
MNG Terminal	ZAKUW2	47.15383	97.62785	2128.000
MNG Terminal	ZAKUW4	46.61646	97.30659	1782.481

Abbreviations: LC, lower constrained; LS, lower single channel; LW, lower wide; UC, upper constrained; UW, Upper wide.
